# A comprehensive clinical evaluation of levocetirizine in the treatment of chronic urticaria in children

**DOI:** 10.3389/fphar.2025.1634089

**Published:** 2025-07-31

**Authors:** Liu Lu, Zhang Xiaodan, Liu Chang, Yan Meixing

**Affiliations:** ^1^ Clinical Pharmacy Department, Qingdao Women and Children’s Hospital, Qingdao, Shandong, China; ^2^ College of Medicine, Ocean University of China, Qingdao, Shandong, China

**Keywords:** levocetirizine, Xyzal, loratadine, children, chronic urticaria, clinical comprehensive evaluation

## Abstract

**Objective:**

Levocetirizine is a second-generation antihistamine that is the first-line drug recommended by the guidelines for the treatment of chronic urticaria in children. However, the current study focused mainly on adults, and a comprehensive evaluation of children has not been reported. Therefore, comprehensive clinical evaluation of levocetirizine in the treatment of chronic urticaria in children is crucial for providing rational clinical drug use and improving the basis of relevant national policies. To conduct a comprehensive clinical evaluation of levocetirizine in the treatment of chronic urticaria in children and provide a reference for rational drug use and related policy decisions in clinical practice.

**Methods:**

A comprehensive clinical evaluation index system for the use of antiallergic drugs in children was established via a literature review, expert interviews, and the Delphi method. Evidence was collected to evaluate the safety, efficiency, economy, suitability, accessibility and innovation of levocetirizine and loratadine for the treatment of chronic urticaria in children.

**Results:**

The comprehensive clinical evaluation index system included six primary indicators, 12 secondary indicators, and 25 tertiary indicators. The total clinical comprehensive evaluation score of levocetirizine was 92.83, whereas that of loratadine was 72.49. The former is superior to the latter in terms of safety, effectiveness, suitability, innovation, and accessibility, whereas the latter is more cost-effective than the former.

**Conclusion:**

The comprehensive clinical value of levocetirizine is greater than that of loratadine in the treatment of chronic urticaria in children, which can provide evidence for the rational use of antiallergic drugs and drug catalog selection in medical institutions.

## 1 Introduction

Levocetirizine, a new second-generation antihistamine, is an active enantiomer of cetirizine, can specifically bind to the peripheral H1 receptor, has rapid and complete oral absorption, has a long half-life, and is not needed for liver metabolism (Hong et al., 2023). Levocetirizine has a wide range of anti-inflammatory effects in addition to selectively antagonizing histamine, which can inhibit the secretion of multiple inflammatory factors (Day et al., 2004). Therefore, levocetirizine has been widely used in the treatment of allergic diseases such as allergic rhinitis and chronic urticaria in children (Zheng et al., 2022; Zhou et al., 2022b). Second-generation antihistamines are recommended by the guidelines as first-line treatments for allergic rhinitis and urticaria in children. Loratadine and levocetirizine belong to this class of drugs, and loratadine tablets are the only ones included in the National Essential Medicines List (2018 edition) (National Health Commission of the People’s Republic of China, 2018; Zhou et al., 2022a). At present, studies have focused mainly on the efficacy and safety of levocetirizine for adult patients (Snidvongs et al., 2017; Jiang, 2021), and a comprehensive clinical evaluation of levocetirizine for chronic urticaria in children has not been reported. Therefore, this study evaluated the clinically comprehensive value of levocetirizine and loratadine in children with chronic urticaria through the establishment of a comprehensive clinical evaluation index system for children’s antiallergic drugs, providing a basis for clinical treatment decision-making to provide a reference for further guidance of rational drugs in pediatric clinical practice.

## 2 Methods

### 2.1 Construction of a clinically comprehensive evaluation system for antiallergic drugs in children

#### 2.1.1 Literature research method

According to the *Technical Guidelines for Clinical Comprehensive Evaluation of Pediatric Drugs (2022 Trial Version)* (National Center for Comprehensive Evaluation of Drugs and Health Technologies, 2022), a systematic search was conducted in Chinese and English databases, including CNKI, VIP Database, China Biology Medicine (CBM) Database, Wanfang Data, PubMed, Embase, and the Cochrane Library, using the following search terms: “levocetirizine,” “loratadine,” “pediatric anti-allergy drug therapy,” “clinical treatment,” “chronic urticaria,” and “clinical comprehensive evaluation” as both subject headings and free-text keywords. Relevant evaluation indicators, such as “treatment rate” and “adverse drug reactions (ADRs),” were extracted and synthesized. By categorizing the retrieved indicators into six main dimensions, namely, “safety, effectiveness, economy, suitability, accessibility and innovation”, a preliminary comprehensive assessment index system was established.

#### 2.1.2 Expert research method and Delphi method

This study employed the Delphi method to conduct consultations focusing on the aforementioned six dimensions, collecting expert opinions through multiple rounds of anonymous correspondence. A clinical comprehensive evaluation expert panel was established, comprising 20 specialists including 17 clinicians and three pharmacists from tertiary and secondary hospitals, to evaluate the proposed indicator system. The questionnaire was distributed online to each expert, and a Likert five-point scale was employed to score the indicators. Higher scores indicated greater importance of the indicators. The experts provided scores and submitted recommendations for adding, modifying, or deleting indicators. The collected feedback was compiled and summarized as reference material, which was then redistributed to all experts for further analysis and judgment. New arguments and opinions were solicited, ultimately yielding a consensus conclusion with high reliability (Wang and Si, 2011).

#### 2.1.3 Analytic hierarchy process (AHP)

Using the AHP method, a pairwise comparison matrix was constructed to evaluate the importance of each factor, and based on this, the relative weights of each factor were determined (Bao et al., 2020). Combined with the above method, a clinical comprehensive evaluation system that is recognized by experts and has guiding significance for the clinical application of children’s anti-allergy drugs was ultimately formed.

### 2.2 Clinical comprehensive evaluation score calculation

In accordance with the preestablished comprehensive clinical evaluation system for anti-allergy drugs in children, data related to the use of levocetirizine and loratadine in the treatment of allergic diseases in children were collected via the literature research method and questionnaire survey method, which provide evidence for comprehensive clinical evaluation. Research studies have focused on relevant research literature, guidelines, expert consensus, and diagnosis and treatment norms. The questionnaire survey investigated the suitability of two kinds of drugs for children by randomly selecting parents of children in the dermatology and otolaryngology departments of our hospital. Moreover, multiple sources of retrieved data, including drug instructions, the website of the State Drug Administration, the website of the FDA, and the Shandong Adverse Drug Reaction Monitoring Center, were used. Twenty experts were once more invited to rigorously score the relevant data gathered for every aspect of drug evaluation via the predetermined scoring standards. The expert score and the weight of each index were used to calculate the overall comprehensive clinical evaluation score. And a radar chart is drawn based on the weights to visually present the evaluation results of the drugs in each dimension.

### 2.3 Statistical method

Statistical analysis was performed using Excel and SPSS 22.0, including matrix operations, mean calculations, t-tests, and χ2 tests. AHP analysis and weight calculations were conducted using Yaahp software, with radar charts generated in Excel.

The expert authority coefficient (Cr) was determined by the coefficient of familiarity degree (Cs) and the coefficient of judgment (Ca), calculated as Cr = (Ca + Cs)/2. In this study, a Cr value ≥0.7 was adopted as the threshold for reliable consultation results.

The Kendall’s coefficient of concordance (Kendall’s W) was employed to assess the degree of agreement among expert opinions, with W ranging from 0 to 1. A higher W value indicates greater consistency among expert assessments. A χ2 test was performed on Kendall’s W, and a P-value <0.05 was considered statistically significant, indicating high reliability of the evaluation results.

## 3 Results

### 3.1 Results of constructing an index system for comprehensive clinical evaluation in children

**FIGURE 1:**
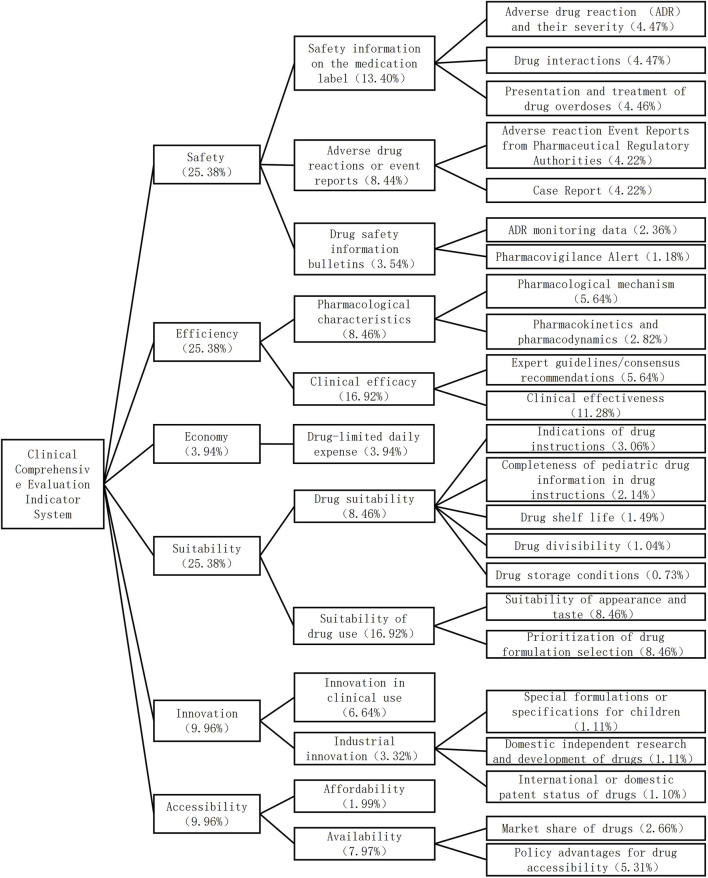
Comprehensive evaluation index system and weight of antiallergic drugs for children.

The comprehensive clinical evaluation index system of antiallergic drugs for children consists of six first-level indices, 12 second-level indices and 25 third-level indices. The first-level indicators include safety, efficacy, economy, suitability, accessibility, and innovation. Among them, the largest and equal weight is given to safety, efficacy, and suitability (25.38%), followed by innovation (9.96%), accessibility (9.96%), and economy (3.94%). The index system and weight reconfiguration are shown in Figure 1.

The two rounds of expert consultation have a 100% effective recovery rate. High authority and trustworthy consultation outcomes were indicated by the experts' authority coefficients in the two rounds, which were 0.830 and 0.725, respectively. The two Kendall coefficients of determination were significantly different (χ2 = 0.319, 0.432, P < 0.01).

### 3.2 Clinical comprehensive evaluation of each dimension indicator evidence

#### 3.2.1 Safety evaluation

##### 3.2.1.1 Safety information on the medication label

###### 3.2.1.1.1 Adverse drug reactions (ADRs) and their severity

Common ADRs associated with levocetirizine and loratadine include headache, drowsiness, and dry mouth. Among rare ADRs, levocetirizine affects mainly the skin system, gastrointestinal system, respiratory system, etc., whereas loratadine affects mainly the ocular organs, cardiovascular system, nervous system, skin system, and hepatobiliary system.

###### 3.2.1.1.2 Drug interactions

Concomitant use of histamine H1 receptor antagonists with central nervous system (CNS) depressants or alcohol may produce additive sedative effects. When co-administered with anticholinergic agents, these antagonists may exhibit enhanced anticholinergic activity. Concurrent use with hepatic enzyme inhibitors may impair drug metabolism, potentially leading to increased cardiotoxic adverse effects such as QT interval prolongation and arrhythmias. Additionally, histamine H1 receptor antagonists may interfere with drug efficacy assessments and mask symptoms of toxicity. Therefore, identifying potential drug-drug interactions (DDIs) is clinically significant.

Currently, no formal DDI studies have been conducted for levocetirizine. However, cetirizine demonstrates no clinically relevant interactions with pseudoephedrine, cimetidine, ketoconazole, erythromycin, azithromycin, glipizide, or diazepam. Food intake may reduce the absorption rate of levocetirizine without affecting its overall bioavailability (Paśko et al., 2017). In contrast, co-administration of loratadine with ketoconazole, macrolide antibiotics, cimetidine, or theophylline may elevate loratadine plasma levels. Food significantly increases the maximum plasma concentration (Cmax) and area under the curve (AUC) of loratadine, though time to maximum concentration (Tmax) remains unaffected (Guo et al., 2025).

###### 3.2.1.1.3 Clinical presentation and treatment of drug overdoses

In cases of levocetirizine overdose, adults may present with drowsiness, whereas in children, they initially show excitement and then develop drowsiness. Symptomatic and supportive treatment should be given for overdose. If the medication has been recently ingested, gastric lavage may be considered, although dialysis cannot completely eliminate the drug. In cases of loratadine overdose, adults may experience symptoms such as headache, tachycardia, and tiredness, whereas children may experience extrapyramidal reactions and palpitations. Vomiting should be induced immediately, and activated charcoal should be used for adsorption for overdose. If vomiting is not successful, the stomach can be washed with normal saline, and catharsis cannot be cleared by hemodialysis (Zhou et al., 2024).

##### 3.2.1.2 Adverse drug reactions or event reports

###### 3.2.1.2.1 Adverse reaction event reports from pharmaceutical regulatory authorities

Pediatric adverse reaction events of levocetirizine and loratadine reported in Shandong Province from 2018 to June 2024 were collected and analyzed. Levocetirizine caused a total of 47 adverse reactions, including one case of severe reaction manifested as general shaking and headache. For loratadine, 119 cases of adverse reactions were reported, with no severe reactions observed. The mean age of children who experienced adverse drug events associated with levocetirizine was 6.77 ± 3.45 years, and most were 3–9 years of age (76.6%). For loratadine, the average age was 7.23 ± 4.13 years, and most patients were 2–7 years of age (51.3%). The adverse reactions caused by levocetirirazine were mostly in the nervous system (74.5%), followed by the gastrointestinal system (19.1%), mental system (4.3%), and urinary system (2.1%). The adverse reactions of the urinary system (enuresis) were not recorded in the manual. The adverse reactions caused by levocetirizine are mostly neurological, accounting for 74.5%, followed by those involving the gastrointestinal system (19.1%), psychiatric system (4.3%), and urinary system (2.1%). Among them, adverse reactions in the urinary system (enuresis) are not documented in the instruction manual. The adverse reactions caused by loratadine are primarily neurological, accounting for 56.3%, followed by those involving the gastrointestinal system (34.5%), skin system (5.0%), respiratory system (3.4%), and tinnitus in the auditory system (0.8%).

###### 3.2.1.2.2 Case report

For additional requirements for specific article types and further information please refer to “Article types” on every Frontiers journal page. This study systematically searched Chinese and English databases and identified a total of 9 cases related to levocetirizine/loratadine ADRs. Among them, one patient was a child, and the remaining eight patients were adults. Levocetirizine was associated with 3 cases, and ADRs included rash pruritus and acute liver injury (Hanna and Bingemann, 2018; Annunziata et al., 2019; Lee et al., 2019). Six patients were treated with loratadine; ADRs involved the immune system, hepatobiliary system, nervous system, gastrointestinal system, etc.,; and one patient died (Bi et al., 2009; Peng, 2007; Jiang, 2021; Wang, 2014; Fei and Xie, 2005; Han, 2006). Loratadine, as a second-generation antihistamine drug with a longer market history, may have accumulated more reported cases. However, a definite case of death was reported, which significantly elevated the overall severity level of reported ADEs for this drug.

##### 3.2.1.3 Drug safety information bulletins

###### 3.2.1.3.1 ADR monitoring data

No safety-related reports concerning levocetirizine or loratadine were identified through retrieval of the National Center for ADR Monitoring website.

###### 3.2.1.3.2 Pharmacovigilance alert

The US FDA has revised the instructions for levocetirizine hydrochloride tablets and oral solutions, adding the following to the adverse reactions section: seizures and febrile convulsions with or without a history of epilepsy; acute generalized exanthemous pustulosis (AGEP); joint pain; movement disorders (including dystonia, oculogyric crisis); convulsions; muscle spasms; and extrapyramidal symptoms. No relevant reports were found for loratadine. There have been no reports of loratadine.

#### 3.2.2 Effectiveness evaluation

##### 3.2.2.1 Pharmacological characteristics

###### 3.2.2.1.1 Pharmacological mechanism

Loratadine is a second-generation H1 antihistamine, and levocetirizine is a novel second-generation H1 antihistamine, both of which have similar mechanisms of action and exhibit no significant anticholinergic or central inhibitory effects. Toxicological studies have not revealed any genetic toxicity or teratogenic effects for either drug.

###### 3.2.2.1.2 Pharmacokinetics and pharmacodynamics

Levocetirizine has a shorter time to peak concentration than loratadine does, with a relatively lower plasma protein binding rate. The plasma elimination half-lives of both methods are similar. Levocetirizine does not undergo a first-pass effect, whereas loratadine does. Levocetirizine exhibits rapid onset of action, providing prompt relief from pruritus and alleviating crying and scratching behaviors in children during allergic episodes, thereby immediately improving quality of life. The absence of a first-pass effect ensures more stable dosing, which is particularly critical for infants and young children with immature hepatic enzyme systems. An average of 85.4% of levocetirizine metabolites are excreted in urine, and 12.9% are excreted in feces. Approximately 80% of loratadine is excreted in equal proportions as a metabolite in both the urine and feces. Levocetirizine is primarily eliminated via renal excretion. However, renal function in children does not reach adult levels until 2 years of age. Therefore, strict weight-based dose adjustment is mandatory when administering levocetirizine to infants and young children. Conversely, loratadine’s dual excretion pathways may confer greater safety in children with immature renal function, provided normal hepatic function is maintained.

##### 3.2.2.2 Clinical efficacy

###### 3.2.2.2.1 Expert guidelines/consensus recommendations

Relevant clinical guidelines from Europe, the United States, Italy, Korea, and China all recommend the use of second-generation unsedating H1 antihistamines as first-line therapy for chronic urticaria in children (Zuberbier et al., 2018; Bernstein et al., 2014; Hide et al., 2012; The Urticaria Research Center of the Dermatology and Venereology Branch of the Chinese Medical Association, 2019; Chow, 2012). Levocetirizine and loratadine both belong to the second-generation non-sedating H_1_ antihistamines.

###### 3.2.2.2.2 Clinical effectiveness

A comprehensive systematic search was conducted in CNKI, VIP Data, the Chinese Biomedical Literature Database, Wanfang Data, PubMed, Embase, and the Cochrane Library to identify randomized controlled trials (RCTs) of levocetirizine *versus* loratadine for meta-analysis. The search time ranged from the establishment of each database to 31 December 2023. A total of 419 pieces of literature were retrieved and ultimately included in one study after screening. This study was a multicenter RCT that included 54 children aged 2–12 years with chronic urticaria, with 26 patients in the levocetirizine group and 28 patients in the loratadine group. The results revealed that the 7-day efficacy rates of levocetirizine and loratadine in treating chronic urticaria in children were 73.08% and 39.29%, respectively, with a statistically significant difference between the two groups (P < 0.05). The effective rates were 73.08% and 50.00% on day 14% and 84.62% and 64.29% on day 28, respectively, with no statistically significant difference between the two groups (P > 0.05). The incidence of adverse reactions in the levocetirizine group was 3.8%, whereas no adverse reactions occurred in the loratadine group. There was no statistically significant difference between the two groups (P > 0.05), and no serious adverse reactions occurred in either group (Wu et al., 2014).

#### 3.2.3 Economic evaluation

Drug-limited daily expenses: This project adopts a cost analysis from the perspective of the healthcare system. Assuming that other medical service costs are basically the same, the direct cost is mainly the drug cost, which is based on the prices from the Shandong Province centralized procurement platform for drugs and medical devices. Since levocetirizine and loratadine have many dosage forms and specifications affecting drug prices, this study uniformly took tablets as an example for calculation. The average daily cost of levocetirizine tablets ranges from 1.07 to 2.14 yuan, whereas that of loratadine is between 0.10 and 0.20 yuan. As for the tablets, the price of loratadine tablets is more favorable than that of levocetirizine tablets.

#### 3.2.4 Suitability evaluation

##### 3.2.4.1 Drug suitability

###### 3.2.4.1.1 Indications of drug instructions

Both drug instructions are suitable for urticaria, allergic rhinitis, and skin pruritus. Additionally, levocetirizine can also be used for eczema and dermatitis, while loratadine can also be used to relieve the signs and symptoms of other allergic diseases.

###### 3.2.4.1.2 Completeness of pediatric drug information in drug instructions

Both drugs indicate the usage and dosage of children over 2 years old. However, the latest guidelines recommend levocetirizine for children aged 6 months and above and loratadine for children aged 2 years and above (Zhou et al., 2022b).

###### 3.2.4.1.3 Drug shelf-life

The shelf-life of different formulations of levocetirizine is 12–24 months, whereas that of loratadine is 18–36 months levocetirizine has a relatively short shelf life. If not used in time, it may expire and be wasted, thereby increasing the cost of medication.

#### 3.2.5 Drug divisibility

Levcetirizine dispersible tablets, ordinary tablets, capsules, and granules, as well as loratadine capsules, orally disintegrated tablets, chewable tablets, and dispersible tablets, have accurate dosages without the need for splitting. However, loratadine ordinary tablets and effervescent tablets require splitting when used for children aged 2–12 years with a weight ≤30 kg. From the perspective of disintegration, the design of levocetirizine is more in line with the needs of children, with accurate dosing, and it also avoids the inconvenience and risks associated with splitting tablets.

#### 3.2.6 Drug storage conditions

The storage conditions of levocetirizine and loratadine are similar. Most formulations require being kept in a closed, dark, or dry place, whereas few require storage in a cool place.

##### 3.2.6.1 Suitability of drug use

###### 3.2.6.1.1 Suitability of appearance and taste

The sugar and flavoring agents (aromatics) in levocetirizine and loratadine syrup, oral solutions, and other formulations are primarily used for taste correction, which can mask unpleasant odors, improve taste, and enhance acceptability (especially for children). In addition, the levocetirizine granule adopts a patented formula and allows it to be taken with milk and juice, thereby facilitating compliance of the child patient.

###### 3.2.6.1.2 Prioritization of drug formulation selection

The marketed dosage forms of levocetirizine include tablets, granules, dispersible tablets, capsules, oral drops, and oral solutions, totaling six types. For loratadine, the marketed dosage forms include tablets, dispersible tablets, chewable tablets, orally disintegrating tablets, effervescent tablets, capsules, granules, and syrups, for a total of eight types. According to a survey of parents, granules are the most preferred formulation for children, accounting for 37.33%, followed by syrup (22.33%), oral solution (16.00%), tablets (12.34%), drops (8.00%), dry suspensions (2.67%), and capsules (1.33%) (Liu et al., 2023). Loratadine has a greater advantage in terms of dosage form diversity, especially directly covering the second most popular syrup formulation in the survey. levocetirizine is available in powder form and oral solution form (with a total acceptance rate of 53.33%), but it is lacking in syrup form. This might have limited its competitiveness in the pediatric market.

#### 3.2.7 Innovative evaluation

##### 3.2.7.1 Innovation in clinical use

Levocetirizine is an active enantiomer of cetirizine, and its pharmacokinetics is linear, with few differences within and between individuals. It is rapidly absorbed after oral administration, does not undergo hepatic metabolism, and has fewer drug interactions. Levocetirizine can be taken with food and is excreted mainly in the urine. Loratadine is also rapidly absorbed orally, and its blood concentration does not change with the dose or administration interval. It exhibits a first-pass effect, is metabolized primarily by cytochrome P450. Coadministration of macrolides, theophylline, cimetidine, and other drugs can increase the blood concentration of loratadine. Taking it with food delays the time to peak concentration but does not affect the peak blood concentration. Although loratadine has a stable blood concentration, its reliance on liver metabolism and the resulting significant risk of drug interactions pose particular challenges that require careful consideration in the pediatric population. On the other hand, levocetirizine’s these characteristics make it a more preferable choice, especially for children who require long-term medication, have concurrent medications, or have difficulties with administration.

##### 3.2.7.2 Industrial innovation

###### 3.2.7.2.1 Special formulations or specifications for children

Both are not specifically designed for children, but levocetirizine granules, oral drops, oral solutions, loratadine dispersible tablets, chewable tablets, effervescent tablets, and syrups are suitable for young children.

###### 3.2.7.2.2 Domestic independent research and development of drugs

Both levocetirizine and loratadine are domestically produced generic drugs. This means that they are produced domestically, with a relatively stable supply chain, and their costs are usually lower than those of original research imported drugs. They are also more accessible, thereby reducing the economic burden on children’s medication.

###### 3.2.7.2.3 International or domestic patent status of drugs

Levocetirizine has 17 dosage form patents. Loratadine has 26 dosage form patents and one utility model patent. The total number of patents for loratadine (27) is significantly higher than that for levocetirizine (17), suggesting that it may be more active or extensive in terms of intellectual property layout and the development of formulations or supporting technologies.

#### 3.2.8 Accessibility evaluation

##### 3.2.8.1 Affordability

Based on a 30-day treatment course for chronic urticaria in children, the proportion of levocetirizine single-course cost in the *per capita* healthcare expenditure of residents in Shandong Province in 2022 (2015 yuan) is 1.49%, and that of loratats is 0.15%. Therefore, for the residents of Shandong Province, the cost burden of using loratadine for treating children’s chronic urticaria is significantly lower than that of levocetirizine.

##### 3.2.8.2 Availability

###### 3.2.8.2.1 Market share of drugs

Taking data from the Minenet platform in 2022 as an example, the sales of levocetirizine in city public hospitals reached 207.46 million yuan, with a market share of 6.95%, whereas the sales of loratadine reached 154.65 million yuan, with a market share of 5.18%. Among the top 20 oral antihistamines before 2022, levocetirizine hydrochloride oral solution occupied 2.24% of the market, levocetirizine hydrochloride granules occupied 1.50%, loratadine tablets occupied 1.35%, and loratadine syrup occupied 1.05%. The findings indicate that in public hospital settings, levocetirizine exhibits superior availability compared to loratadine, suggesting a prescribing preference among physicians or procurement inclination toward levocetirizine. And both the oral solution (2.24%) and the granules (1.50%) are child-friendly formulations, making them more suitable for younger patients. This indirectly indicates that levocetirizine has a stronger pediatric compatibility.

###### 3.2.8.2.2 Policy advantages for drug accessibility

Levocetirizine has been included in the “National Basic Medical Insurance, Work-related Injury Insurance, and Maternity Insurance Drug Catalog” (2023 version); drops, tablets, capsules, and dispersible tablets are class B national medical insurance; and levocetirizine tablets have entered the volume-based procurement catalog. Loratadine tablets and capsules have been included in the “National Basic Drug Directory” (2018 version); except for the syrup in medical insurance class B, other dosage forms of loratadine, such as capsules, tablets, and dispersible tablets, are classified as medical insurance class A; loratadine tablets have also been included in the volume-based procurement catalog. Loratadine has significant advantages in terms of policy coverage. This means that it is more accessible and less costly for children, especially those seeking medical treatment at the grassroots level. Levocetirizine provides an important pediatric formulation. Its oral drops have been included in the medical insurance category B, offering an important and convenient treatment option for infants and young children.

### 3.3 Comprehensive clinical evaluation scoring

**FIGURE 2:**
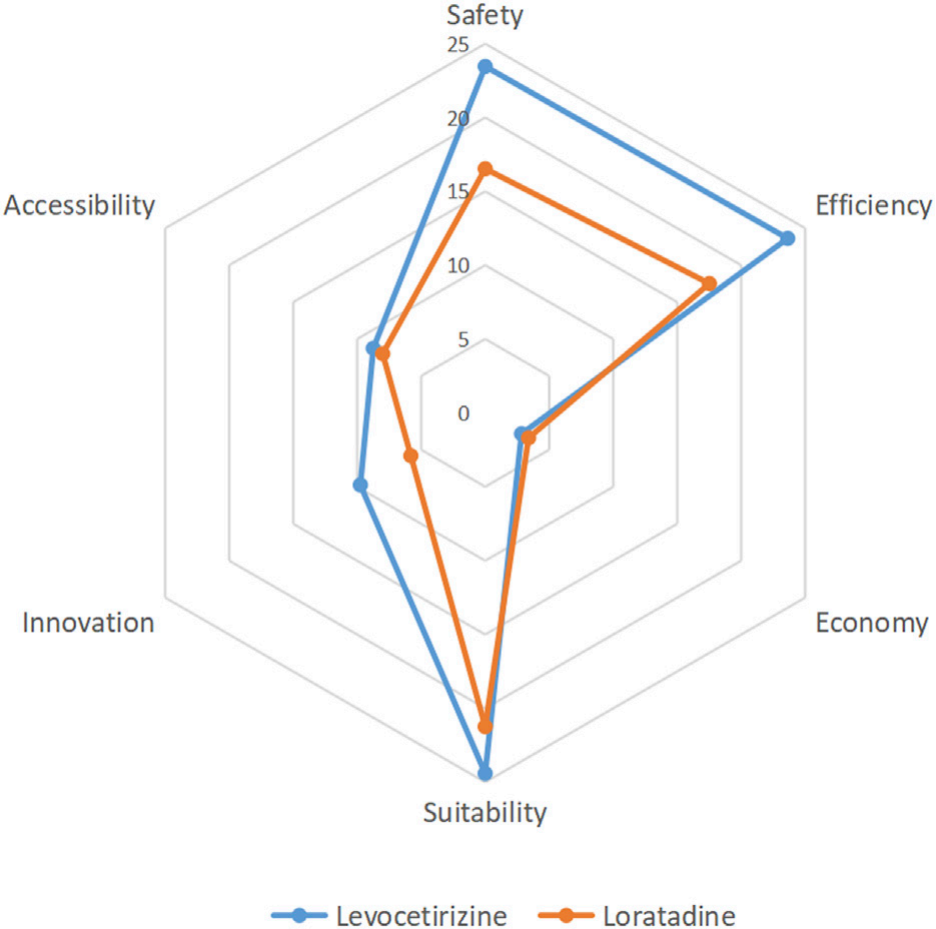
A comprehensive clinical evaluation revealed that levocetirizine has significant advantages over lororeatadine in terms of safety, efficacy, suitability and innovation and has high comprehensive clinical value.

Based on the comprehensive evaluation evidence from multiple dimensions, a panel of 20 experts independently scored the tertiary indicators for both levocetirizine and loratadine. The mean values of expert ratings for each indicator were calculated, and corresponding scores were derived by incorporating indicator weights. The summation of all tertiary indicator scores yielded the total clinical comprehensive evaluation score for each drug. Radar charts were generated based on primary indicator scores (Figure 2). The results demonstrated that levocetirizine exhibited superior performance over loratadine across four key dimensions: safety, efficacy, suitability, and innovation, indicating higher overall clinical value. The aggregated scores revealed that levocetirizine achieved a clinical comprehensive evaluation score of 92.83, whereas loratadine scored 72.49. Based on the t-distribution calculation, the 95% confidence intervals for the two are (66.00–119.67) and (51.88–93.10) respectively, and there is no overlap between the confidence intervals. This indicates that levocetirizine has more significant clinical application advantages.

## 4 Discussion

Chronic urticaria has a duration of more than 6 weeks and is prone to recurrent attacks, which significantly affect the learning and daily lives of children and patients and reduce their quality of life. This study establishes a comprehensive clinical evaluation system for children receiving anti-allergic drugs through literature research, expert surveys, the Delphi method, and the analytic hierarchy process. Taking levocetirizine and loratadine as first-line treatment drugs for children with chronic urticaria as examples, a comprehensive clinical evaluation was conducted from six dimensions, including safety, efficacy, economy, suitability, innovativeness, and accessibility, which can provides a decision-making basis for the rational use of pediatric anti-allergy drugs in medical institutions and the selection of drug directories.

The results of the comprehensive evaluation indicate that levocetirizine has greater comprehensive clinical value than loratadine does. In addition to its economic value, levocetirizine is superior to loratadine in terms of safety, efficacy, suitability, and innovation. In terms of safety, data from the ADR monitoring platform and case reports in Shandong Province revealed that the overall number of ADRs associated with levocetirizine was lower than that associated with loratadine, and serious adverse reactions resulting in death were reported for loratadine, whereas no fatal cases were reported for levocetirizine. In terms of efficacy, levocetirizine and loratadine have similar pharmacological properties, but levocetirizine does not have a first-pass effect. Multicenter RCTs have shown that the treatment efficacy of levocetirizine is slightly greater than that of loratadine, but the difference was not significant. In terms of the economy, the limited daily drug cost of levocetirizine is slightly greater than that of loratadine. In terms of suitability, levocetirizine has a wider range of application than loratadine does, and its appearance and taste are more suitable for children. However, it has a shorter shelf life. Both drugs have child-friendly dosage forms. In terms of innovation, levocetirizine granules use a patented lactose formula, but the number of relevant domestic patents is less than that of loratadine. In terms of accessibility, although most dosage forms of levocetirizine have entered the medical insurance category B list, they are not included in the national essential medicine list. This results in levocetirizine having a weaker policy advantage and lower affordability than loratadine does, but it has a higher market share than loratadine does.

This study has certain limitations. For example, the consulted experts are primarily from the clinical field; experts from epidemiology, health economics, and other related fields could be included to make the evaluation results more scientific and comprehensive in the future. Second, only one multicenter RCT was included in the efficacy evaluation, and thus, the conclusions drawn are preliminary and exploratory due to the limited sample size of a single RCT. From a professional perspective, research on pharmacological interventions for chronic urticaria in children faces multiple inherent barriers. First, ethical review is significantly stricter than that for adult studies, and parental acceptance of the control group is low. Second, pharmaceutical companies lack sufficient motivation for development, given the relatively small market share of pediatric indications. More critically, both levocetirizine and loratadine are off-patent medications, and manufacturers are reluctant to invest in head-to-head trials. Therefore, future studies should employ large-scale, rigorously designed RCTs to further validate these findings. Moreover, during the evidence collection process, a search of the adverse drug reaction monitoring network and other sources revealed that there is a scarcity of real-world data related to the child population. There is a need to conduct further prospective multicenter real-world studies to provide more evidence for the clinically rational use of levocetirizine.

## 5 Conclusion

There are many kinds of second-generation H1 antihistamines, few clinical guidelines and consensus recommendations, and lack of comprehensive clinical evaluation results. By comparing the comprehensive clinical evaluation of levocetirizine and loratadine in the treatment of chronic urticaria in children, we found that levocetirizine has comprehensive clinical value compared with loratadine in terms of safety, efficacy, economy, applicability, accessibility and innovation. Through the clinical comprehensive evaluation of levocetirizine, we provided a basis for rational clinical use of anti-allergy drugs and related policies, which can help clinical optimization of drug use, more accurate control of drug use, avoid unnecessary drug waste, and thus improve medical quality.

## Data Availability

The original contributions presented in the study are included in the article/supplementary material, further inquiries can be directed to the corresponding author.
